# Predictive value of a stemness-based classifier for prognosis and immunotherapy response of hepatocellular carcinoma based on bioinformatics and machine-learning strategies

**DOI:** 10.3389/fimmu.2024.1244392

**Published:** 2024-04-17

**Authors:** Erbao Chen, Zhilin Zou, Rongyue Wang, Jie Liu, Zhen Peng, Zhe Gan, Zewei Lin, Jikui Liu

**Affiliations:** ^1^ Department of Hepatobiliary and Pancreatic Surgery, Peking University Shenzhen Hospital, Shenzhen, Guangdong, China; ^2^ Department of Ophthalmology, Affiliated Eye Hospital of Wenzhou Medical University, Wenzhou, Zhejiang, China

**Keywords:** hepatocellular carcinoma, prognostic signature, stemness, tumor microenvironment, immunotherapy response

## Abstract

**Objective:**

Significant advancements have been made in hepatocellular carcinoma (HCC) therapeutics, such as immunotherapy for treating patients with HCC. However, there is a lack of reliable biomarkers for predicting the response of patients to therapy, which continues to be challenging. Cancer stem cells (CSCs) are involved in the oncogenesis, drug resistance, and invasion, as well as metastasis of HCC cells. Therefore, in this study, we aimed to create an mRNA expression-based stemness index (mRNAsi) model to predict the response of patients with HCC to immunotherapy.

**Methods:**

We retrieved gene expression and clinical data of patients with HCC from the GSE14520 dataset and the Cancer Genome Atlas (TCGA) database. Next, we used the “one-class logistic regression (OCLR)” algorithm to obtain the mRNAsi of patients with HCC. We performed “unsupervised consensus clustering” to classify patients with HCC based on the mRNAsi scores and stemness subtypes. The relationships between the mRNAsi model, clinicopathological features, and genetic profiles of patients were compared using various bioinformatic methods. We screened for differentially expressed genes to establish a stemness-based classifier for predicting the patient’s prognosis. Next, we determined the effect of risk scores on the tumor immune microenvironment (TIME) and the response of patients to immune checkpoint blockade (ICB). Finally, we used qRT-PCR to investigate gene expression in patients with HCC.

**Results:**

We screened CSC-related genes using various bioinformatics tools in patients from the TCGA-LIHC cohort. We constructed a stemness classifier based on a nine-gene (*PPARGC1A, FTCD, CFHR3, MAGEA6, CXCL8, CABYR, EPO, HMMR*, and *UCK2*) signature for predicting the patient’s prognosis and response to ICBs. Further, the model was validated in an independent GSE14520 dataset and performed well. Our model could predict the status of TIME, immunogenomic expressions, congenic pathway, and response to chemotherapy drugs. Furthermore, a significant increase in the proportion of infiltrating macrophages, Treg cells, and immune checkpoints was observed in patients in the high-risk group. In addition, tumor cells in patients with high mRNAsi scores could escape immune surveillance. Finally, we observed that the constructed model had a good expression in the clinical samples. The HCC tumor size and UCK2 genes expression were significantly alleviated and decreased, respectively, by treatments of anti-PD1 antibody. We also found knockdown UCK2 changed expressions of immune genes in HCC cell lines.

**Conclusion:**

The novel stemness-related model could predict the prognosis of patients and aid in creating personalized immuno- and targeted therapy for patients in HCC.

## 1 Introduction

Cancer stem cells (CSCs) are an undifferentiated subset of cells with an indefinite self-renewal ability (1). Studies have shown the significant involvement of CSCs in tumorigenesis, cell metastasis, and drug resistance; hence, CSCs could indicate poor prognosis in cancer patients (2). Furthermore, CSCs promote the progression of hepatocellular carcinoma (HCC) by inducing genetic, proteomic epigenetic, and transcriptomic changes (3). In addition, CSCs increase the incidences of metastasis and drug resistance in HCC. The mRNA expression-based stemness index (mRNAsi) is used to quantitatively measure the degree of similarity between CSCs and cancer cells. High mRNAsi scores suggest enhanced biological activity of CSCs and highly aggressive tumor dedifferentiation, characterized by histopathological grades (4). Furthermore, studies have demonstrated that mRNAsi could predict cancer recurrence and drug resistance (5). However, the underlying mechanism and the clinical significance of mRNAsi in HCC remain largely unknown. Therefore, additional studies on CSCs and immune status are required to improve the survival outcomes and therapeutic efficacy in patients with HCC.

Several studies have extensively focused on immunotherapy for cancer therapeutics and could benefit patients with advanced cancers. Atezolizumab (anti-PD-L1 antibody) plus bevacizumab (anti-VEGF antibody) combination therapy has demonstrated superior efficacy in improving the patient’s prognosis compared to standard sorafenib-based therapy (6). Hence, this combination therapy is used as a first-line therapy for cancer treatment globally (7). This combination therapy inhibits angiogenesis, reduces the infiltration of myeloid-derived suppressor cells (MDSCs), adjusts the M1/M2 tumor-associated macrophage ratio, and promotes the infiltration and functional recovery of CD8+ T cells (8). This could be the underlying mechanism of positive outcomes of anti-PD-L1 immunotherapy in patients with HCC. However, there are several unanswered questions regarding immunotherapy in HCC. First, studies are necessary to identify drugs that could increase the anti-angiogenic efficacy of immune-checkpoint blockade (ICB). Second, the effectiveness of immunotherapeutic strategies, such as adoptive T cell transfer, ICBs, and vaccinations for patients with HCC is still unclear. Last, biomarkers for identifying, developing, and predicting patients’ response to ICBs is still missing.

We obtained mRNAsi of patients with HCC based on transcriptomic data. Next, we developed a unique prognostic signature based on these mRNAsi scores to classify patients into three stemness subtypes. These subtypes had distinct clinical features, functional enrichment, and tumor mutational burden (TMB). Next, we validated the ability of the stemness-based classifier to predict the patient’s prognosis on patients from the Gene Expression Omnibus (GEO) database. We performed integrated bioinformatic analysis to evaluate the ability of the mRNAsi model to predict the patient’s clinical characteristics, microenvironment features, genetic patterns, and drug response. Finally, we used machine learning algorithms to construct a stemness classifier based on the expression of nine genes to determine the status of mRNAsi and predict the patient’s response to chemo- and immunotherapy.

## 2 Materials and methods

### 2.1 Data processing

The gene expression data of patients with HCC were obtained from “the Cancer Genome Atlas Liver Hepatocellular Carcinoma” (TCGA-LIHC; https://portal.gdc.cancer.gov/projects/TCGA-LIHC) and the GEO (https://www.ncbi.nlm.nih.gov/geo/) databases. Patients without complete information were excluded. Finally, we selected 365 and 221 patients from TCGA (9) and GSE14520, respectively, and GSE14520 was used for external validation. Since data were obtained from two databases, to ensure the data can be compared and reduce batch effects, transcript per million values were log2 transformed and normalized using the R package. We determined the correlation between gene signatures and the response of patients from the ERP117672 and GSE202069 cohorts to immunotherapy.

### 2.2 Calculating mRNAsi

We constructed the mRNAsi model using the “one-class logistic regression (OCLR)” algorithm (5). Next, we used “Spearman correlation analysis” to determine the similarity between cancer cells of patients with HCC and stem cells. Finally, we determined the mRNAsi by calculating the correlation coefficient value between 0-1. A stemness index closer to 1 indicates a higher stemness capacity.

### 2.3 Identification of mRNAsi-related differentially expressed genes

The optimal cut-off value for the calculated mRNAsi score was determined using the “survminer” package. Next, patients with HCC from the TCGA-LIHC cohort were categorized into high- and low-mRNAsi subgroups based on this cut-off value. We used the “linear models for microarray data (limma)” package to screen for mRNAsi-related DEGs in tumor and normal tissues in both databases (10) based on the following criteria: “|R| > 0.3” and “*P* < 0.05.” Next, the “false discovery rate” (FDR) method was used for correcting the results. Finally, we identified 19 mRNAsi-related DEGs for predicting prognosis using the “univariate Cox regression analysis.”

### 2.4 Determining the stemness-based classification

We performed “unsupervised Consensus clustering” using the “ConsensusClusterPlus” R package to determine a stemness-based classification (11). First, we employed the “k-means algorithm” for subsampling the items at 80% and categorizing them into various groups. The process was repeated 500 times. Next, we used the “consensus matrix” and “cumulative distribution function” plots for determining the optimal cluster numbers. The overall survival (OS) of patients in the stemness subtypes was determined using Kaplan-Meier (KM) survival curve analysis. Additionally, we employed the “limma” package to identify DEGs with “|log2 fold change (FC) |>1” and “*P* < 0.05” in the three subgroups. Finally, we performed “functional enrichment analysis” using the “WebGestaltR” package on these DEGs.

### 2.5 Identification of the characteristics of immune cells infiltrating tumors of patients with HCC

To determine the relative abundance of immune cell types in a mixed cell population in patients with HCC, we imported and analyzed the unnormalized RNA-Seq data using the “Cell-type Identification by Estimating Relative Subsets of RNA Transcripts (CIBERSORT)” tool (12). Next, we predicted the infiltration of stromal/immune cells in patients with HCC using the “Estimation of STromal and Immune cells in MAlignant Tumor tissues using Expression data (ESTIMATE)” algorithm (13). The “ESTIMATE” algorithm calculates the stromal, immune, and ESTIMATE scores, which show stromal abundance, immune cell infiltration, and tumor purity, respectively. We performed “single sample Gene Set Enrichment Analysis (ssGSEA)” using the “Gene set variation analysis (GSVA)” R package and assessed 29 immune gene signatures to quantify the enrichment scores of immune-related terms. Finally, we analyzed the expression of several immune checkpoint genes to determine the response of patients in the two stemness subtypes to immunotherapy.

### 2.6 GSVA

We performed an unsupervised “GSVA” R package to determine the differential activity of pathways in patients in the two stemness subtypes. The “h.all.v7.4.symbols.gmt” and “c2.cp.kegg.v7.4.symbols.gmt” gene sets were retrieved from the “Molecular Signatures database” (14) and were selected as the background gene set. In addition, the “limma” R package was used to perform differential analysis of the “Kyoto Encyclopedia of Genes and Genomes (KEGG)” and “HALLMARK” pathways in patients in the two stemness subtypes. The significantly enriched pathways were identified based on the following criteria: “FDR < 0.05” and “log2 FC > 1.”

### 2.7 Several machine learning algorithms were used to construct and verify the stemness-based classifier

We used “univariate Cox regression analysis” to analyze 816 DEGs in the three subgroups. Genes with “*P* < 0.001” and “hazard ratio (HR)> 1” were classified as risk genes, and genes with “*P* < 0.001” and “HR < 1” were classified as protective genes. To accurately predict the status of the stemness subtype, we ranked risk and protective genes based on importance using “the least absolute shrinkage and selection operator (LASSO) regression analysis” via the “glmnet” R package. “Multivariate logistic regression analysis” was used for constructing a stemness-based classifier based on the following formula: riskscore = Coefficient (stemness gene i) * Expression (Stemness gene i). Next, the classifier was normalized to the 0-1 range. We calculated the normalized riskscore using the “survminer” package, and the patients were categorized based on the threshold value of the risk score into the high-risk group (HRG) and the low-risk group (LRG). Finally, we used the “time-dependent receiver operating characteristic (ROC) curve” to determine the predictive ability of the stemness-based classifier model, and the area under the ROC curve (AUC) value was determined using the “pROC” package. We also analyzed the differences in tumor immune microenvironment (TIME) and the pathways enriched in patients in the HRG and LRG.

### 2.8 Prediction of chemotherapy

We obtained data from the “Cancer Cell Line Encyclopedia” database and used the “pRRophetic” software to predict the patient’s response to erlotinib by determining the half-maximal inhibitory concentration (IC_50_) value of the drug for all patients (15, 16). The IC_50_ value is the measure of the drug efficacy in suppressing a specific biological function in patients with HCC. Therefore, a lower IC_50_ value indicates higher sensitivity of patients to a specific drug. We used the “ridge regression” model on the expression data of patients with HCC to predict the response of patients, and the accuracy of the prediction was evaluated using 5-fold cross-validation.

### 2.9 Cell lines and culture, transfection

Human hepatoma cell lines including MHCC-97H, PLC/PRF/5, Huh7, HepG2, and Hep3B as well as Hepa1–6 cell line (C57BL/6‐derived hepatoma) were all commercially obtained from Cell Bank of Type Culture Collection of the Chinese Academy of Sciences. DMEM or RPMI‐1640 media (Gibco, Thermo Fisher Scientific, Waltham, MA, USA) added with 1% penicillin and streptomycin (Solarbio, Beijing, China) and 10% FBS (Biological Industries, CT, USA) was used to grow the above mentioned cell lines, which were incubated in a 5% CO_2_ incubator at 37 °C. With the use of Lipofectamine 2000 (Invitrogen, Carlsbad, CA, USA), the sequences of human UCK2 siRNAs were transfected into hepatoma cell lines.

### 2.10 Experimental mouse models

A total of 5x10^6^ Hepa1-6 cells in Matrigel were subcutaneously implanted into 5-week-old male C57BL/6 mice (Beijing HFK Bioscience) via the right flank to develop immunocompetent murine models of HCC. When tumors grew to a volume of 200-300 mm^3^, the mice were given PBS or Anti-PD1 (Hamster anti-murine PD1 mAb J43 (BioXCell) i.p. at 10 mg/kg every 3 days for a total of 5 doses) in a randomized manner. Finally, the mice tumors were collected for further studies.

### 2.11 RNA isolation, real−time quantitative reverse transcription polymerase chain reaction, and clinical samples

We performed RNA isolation and qRT-PCR as described previously (17). **Supplementary Table S1** shows the primer sequences. The study protocol was approved by the Ethics Committee of Peking University Shenzhen Hospital. Informed consent was obtained from all patients included in the study.

### 2.12 Statistical analysis

We used the “Pearson correlation” test to determine the correlation between two variables that are not linearly correlated. The categorical and pairwise characteristics of different subgroups were compared using the “Chi-squared test”. The data of skewed distribution and ordinal data in the subgroups were compared using the “Wilcoxon test”. Student t test was used to compared between two groups. We performed a “KM survival curve” analysis to determine the OS of patients with HCC for a specific period. The significant difference in survival was determined using the “log-rank” test. We used the “R (version 4.2.0)” software for statistically analyzing the data. *P* < 0.05 (two-tailed) was considered statistically significant.

## 3 Results

### 3.1 Correlation between mRNAsi and clinicopathological features of patients with HCC

First, we used the “OCLR” algorithm on the transcriptomic data of patients in TCGA-LIHC cohort to obtain the mRNAsi scores. Next, we categorized patients into the mRNAsi-high and mRNAsi-low groups based on the optimal cutoff value calculated using the “survminer,” Next, we performed the “KM survival curve analysis” to determine the impact of mRNAsi on the OS of patients with HCC. The KM plot showed that the OS of patients in the mRNAsi-high group was poor compared to the mRNAsi-low group (**Supplementary Figure 1A**). Next, we ranked all patients from low to high mRNAsi scores and determined the correlation between the mRNAsi scores and the clinicopathological features of patients. A significant difference in gender, survival status, and tumor grade was observed in patients in both mRNAsi groups (**Supplementary Figure 1B**). Additionally, the mRNAsi scores of patients with stage II HCC were significantly higher compared to stage I HCC; however, no such trend was observed in patients with more advanced clinical stages (**Supplementary Figure 1C**). Moreover, the OS of patients with stage I and II HCC in the mRNAsi-high group was poor compared to the mRNAsi-low group (**Supplementary Figure 1D**). We observed a similar trend in patients with stage III and IV HCC; however, the difference was not significant (**Supplementary Figure 1E**). There was a trend between mRNAsi score and tumor grade (**Supplementary Figure 1F**). The OS of patients with grade I and II HCC in the mRNAsi-high group was better compared to the mRNAsi-low group (**Supplementary Figure 1G**). Similarly, the patients with stages III and IV exhibited a significant trend (**Supplementary Figure 1H**).

### 3.2 Identifying stemness subtypes using mRNAsi-related DEGs

**Figure 1:**
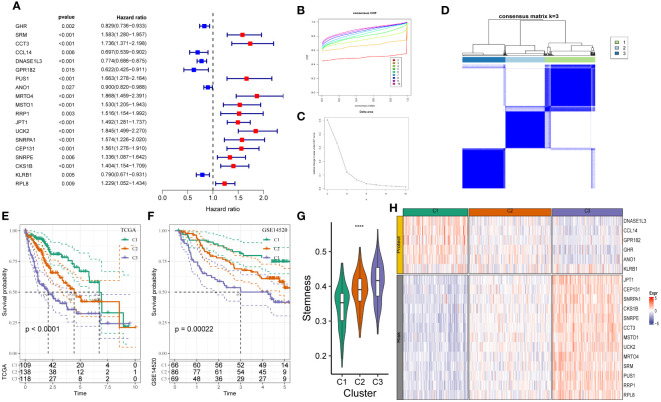
Construction of stemness subtypes with distinct functional annotation and survival outcomes. **(A)** Results for consensus clustering based on the expression patterns of 19 stemness biomarkers. K–M survival analyses indicated significantly worse OS in the high mRNAsi groups. **(B, C)** The optimal number of clusters was determined using CDF and the area under the CDF curve as three. **(D)** The distribution of different clusters with the index k = 3. **(E, F)** Kaplan-Meier survival analysis showed the significantly different survival rates between three stemness subtypes in **(E)** TCGA-LIHC cohort and **(F)** GSE14520 cohort. **(G)** The boxplot showed that the different stemness score between three subtypes in TCGA-LIHC cohort. **(H)** The heatmap of the expression patterns of mRNAsi-related genes between three subgroups. ****, P<0.0001.

We constructed a novel stemness-based classifier using multiple bioinformatic tools to investigate the mechanism of the two mRNAsi groups and the functions of the mRNAsi model. First, we identified DEGs in tumor and adjacent tissues of patients in TCGA-LIHC and GSE14520 cohorts based on the following criteria: “∣FC∣> 1.2” and “*P* < 0.05.” Next, we performed the correlation analysis on DEGs and the mRNAsi scores using “Pearson’s correlation coefficient” based on the following criteria “∣R∣> 0.3” and “*P* < 0.05.” We identified 19 mRNAsi-related DEGs, of which 13 genes were upregulated (risk genes, HR > 1) and six genes were downregulated (protective genes, HR < 1) in patients in the mRNAsi-high group (**Figure 1A**).

We determined the heterogeneity of stemness characteristics by constructing a novel stemness-based classifier based on 19 mRNAsi-related DEG expressions using “unsupervised consensus clustering.” The patients were categorized into the stemness subtype C1 (109 cases, 29.9%), the stemness subtype C2 (138 cases, 37.8%), and the stemness subtype C3 (118 cases, 32.3%). **Figures 1B–D** show a significant difference in the expression pattern of 19 mRNAsi-related DEGs. Furthermore, the “KM survival curve” analysis showed that the patients in C3 had a significantly poor prognosis compared to patients in C1 and C2 (**Figure 1E**) in TCGA-LIHC cohort. Similar results were observed in the GSE14520 cohort (**Figure 1F**). The mRNAsi scores of patients in C3 were higher compared to C1 and C2 (**Figure 1G**). **Figure 1H** shows an increase in *JPT1, CEP131, SNRPA1, CKS1B, SNRPE, CCT3, MSTO1, UCK2, MRTO4, SRM, PUS1, RRP1*, and *RPL8* expression (risk genes) in patients in C3. Furthermore, an increase in *DNASE1L3, CCL14, GPR182, GHR, ANO1*, and *KLRB1* (protective genes) expression was observed in patients in C3 (**Figure 1H**). These results showed that the stemness-based classifier could predict the patient’s prognosis and should be analyzed further.

### 3.3 Relationship between stemness subtypes and clinical features

We determined the clinical features of patients in the three stemness subtypes. The percentage of younger patients in C3 was high (**Supplementary Figure 2A**). The results revealed no significant difference in the gender of patients from TCGA-LIHC cohort in the three stemness subtypes (**Supplementary Figure 2B**). Additionally, a significant difference in the clinical features, such as tumor stage, grade, and stemness, was observed in patients in the three stemness subtypes (**Supplementary Figures 2C-E**). The patients with high grades were classified in C3, which could be the cause of the poor prognosis of patients in C3 (**Supplementary Figure 2F**).

### 3.4 Stemness subtypes showed distinct DNA damage and TMB

DNA damage assessment, including aneuploidy, homologous recombination deficiency (HRD), the fractions of genome altered, and the number of segments, is associated with malignancy and immune cell infiltration. Aneuploidy occurs in various cancers and indicates an increased evasion of immune surveillance by cancer cells and reduced patient response to immunotherapy (18). The aneuploidy score of patients in C3 was significantly higher compared to C1 and C2 (**Supplementary Figure 3A**). Furthermore, the incidences of HRD were higher in patients in C3 compared to C1 and C2 (**Supplementary Figure 3B**). Furthermore, the fractions of genome altered were lower in patients in C1 and C2 compared to C3 (**Supplementary Figure 3C**). The number of segments in patients in C1 was low compared to C2 (**Supplementary Figure 3D**). A significant difference was observed in the rate of TMB in patients in C3 and C1 (**Supplementary Figure 3E**). We also compared our signature to a previously published classifier. We obtained the HCC subtypes from a previous study (19). The study showed that the prognosis of patients in C3 was better (19), and these patients were primarily enriched in C1 in our study (**Supplementary Figure 3F**). TMB score of patients in C3 was significantly higher compared to C1. The rate of mutations in *TP53*, *CSMD3*, *LRP1B*, and *DNAH7* was lower in patients C3 compared to C1 (**Supplementary Figure 3G**). The rate of mutations in *TP53* (50% vs. 24%, 11%), *CSMD3* (14% vs. 6%, 4%), *LRP1B* (9% vs. 12%, 2%), and *DNAH7* (10% vs. 7%, 2%) was higher in patients in C2. Therefore, the degree of malignancy in patients in C3 was higher.

### 3.5 Immune status of patients in different stemness subtypes was different

**Figure 2:**
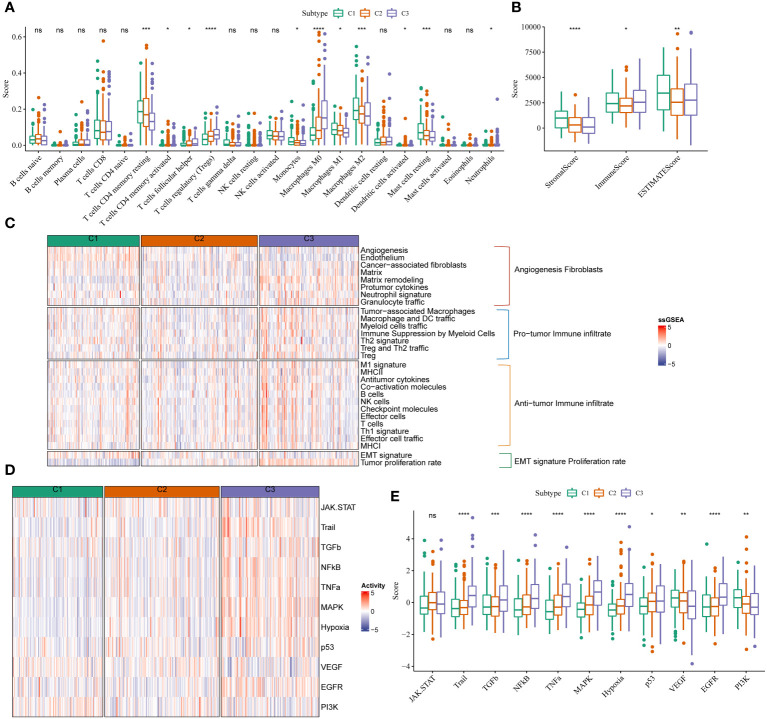
mRNAsi scores were correlated with different TIME patterns of HCC patients. **(A)** Comparisons of the abundances of 22 immune cells in three clusters by CIBERSORT. **(B)** Comparisons of stromal score, immune score, and ESTIMATE score between three clusters. **(C)** Comparisons of the abundances of 29 gene signatures in three clusters. **(D, E)** Comparisons of the 11 oncogenic pathways in three clusters by PROGENy algorithm. *P<0.05, **P<0.01, ***P<0.001, ****P<0.0001, ns, Not Significant.

We analyzed the abundance of immune cell infiltration in patients from TCGA-LIHC cohort using CIBERSORT (**Figure 2A**). A difference in the abundance of macrophages was observed in patients in these three stemness subtypes. A significant increase in the abundance of M0 and a significant decrease in M1 and M2 macrophage abundance was observed in patients from TCGA-LIHC cohort in C3. Additionally, the results revealed a significant decrease in the abundance of resting mast cells in patients in C3 compared to C1. A significant decrease in the abundance of resting memory CD4 cells, which are activated T cells, was observed in patients from TCGA-LIHC cohort in C3. Boxplots were used to explore the relationship between the three immune cell clusters based on ssGSEA and mRNAsi scores. The results showed low stromal scores and a high immune score in patients in C3 (**Figure 2B**). We used ssGSEA to assess 29 immune gene signatures concluding the primary function and immune, stromal, and other cell (20). The infiltration of pro and anti-tumor immune cells was high in patients in C3. Moreover, the proliferation of cancer cells in patients in C3 was high (**Figure 2C**). The pro and anti-tumor immune score of patients in C3 was high. Finally, we performed PROGENy analysis (21) to identify enriched signaling pathways. The results revealed that the Trail, TGF-β, NFKB, TNFA, MAPK, HYPOXIA, P53, and EGFR signaling pathways were enriched in patients in C3, and the VEGF and PI3K signaling pathways were enriched in patients in C1 (**Figures 2D, E**).

### 3.6 Predictive value of the immunotherapy response and targeted therapy of different stemness subtypes

**Figure 3:**
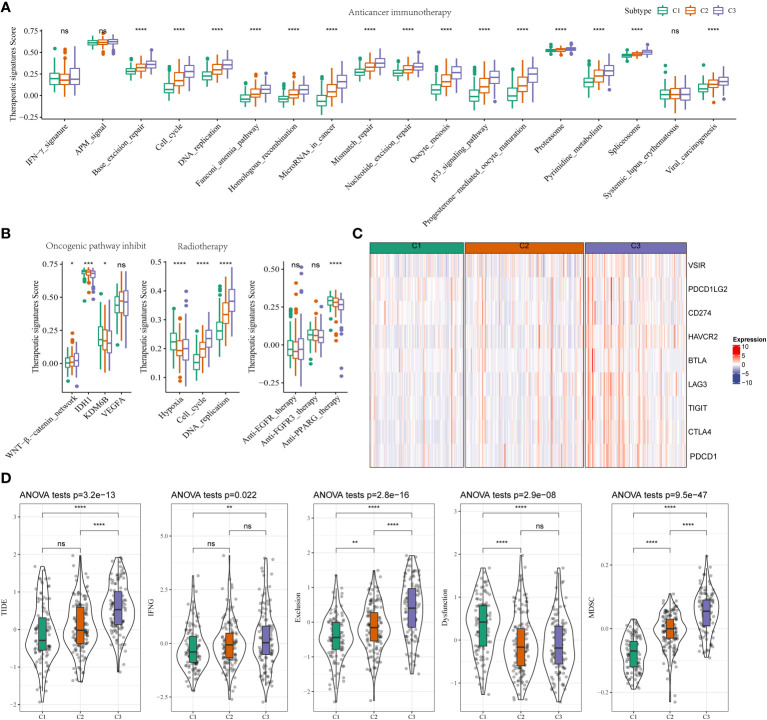
Different TIME status, immunogenomic patterns and sensitivity to targeted therapy between two stemness subtypes **(A)** Differences in the enrichment scores of immunotherapy-predicted pathways between high- and low-risk subgroups in TCGA-LIHC cohort. **(B)** Comparisons of oncogenic pathway inhibit, radiotherapy, anti-EGFR, and anti-FGFR3, and anti-PPARG therapy in the three clusters. **(C)** Expression status of VSIR, PDCD1LG2, CD274, HAVCR2(TIM-3), BTLA, LAG-3, TIGIT, CTLA-4, and PDCD1 (PD-1) in three clusters. **(D)** Comparisons of TIDE score, IFNG, T cell exclusion dysfunction, and MDSC in the high- and low-risk groups. *P<0.05, **P<0.01, ***P<0.001, ****P<0.0001, ns, Not Significant.

The results showed an increase in therapeutic signatures, such as base excision repair, cell cycle, the Fanconi anemia pathway, DNA replication, miRNA in cancer, nucleotide excision and mismatch repair, homologous recombination, the p53 signaling pathway, oocyte meiosis, oocyte maturation mediated by progesterone, pyrimidine metabolism, proteasome, spliceosomes, antiviral carcinogenesis in patients in C3 compared to C1 and C2 (**Figure 3A**). An increase in the WNT-β-catenin signaling pathway, IDH1, and KDM6B expression was observed in some therapeutic signature scores. We also found that the radiotherapy-related signatures were significantly differentially among hypoxia, cell cycle, and DNA replication. Based on a previous study (22), patients in C3 could benefit from anti-PPARG therapy instead of anti-EGFR and anti-FGFR3 therapies (**Figure 3B**). Immunotherapy is widely used for treating patients with HCC. Hence, we used the HisgAtlas dataset for analyzing immune checkpoint genes. The results revealed a significant increase in immune checkpoint gene expression in C2 compared to C1. An increase in the expression of several immune checkpoint genes, such as *VSIR, PDCD1LG2, CD274, HAVCR2 (TIM-3), BTLA, LAG-3, TIGIT, CTLA-4*, and *PDCD1*, was observed in patients in the high-risk subgroups of C3 (**Figure 3C**). Furthermore, we used the “Tumor Immune Dysfunction and Exclusion (TIDE)” software to determine the response of patients in the two clusters to immunotherapy. Patients with higher TIDE scores could evade immune surveillance, which indicates that these patients may not benefit from immunotherapy. The TIDE scores of patients from TCGA-LIHC cohort in C3 were higher compared to C1 or C2, which suggests that patients in C3 could evade immune surveillance and may not benefit significantly from treatment with immunotherapy (**Figure 3D**). There was an increase in IFN-β, T cell exclusion, and dysfunction, and MDSC scores, and low T cell dysfunction scores in patients in C3 (**Figure 3D**). This could be the underlying cause of the high degree of malignancy in patients in C3.

### 3.7 DEGs and pathways in different stemness subtypes

First, we used the “limma” package to identify DEGs in these three stemness clusters. We identified 166 upregulated and 303 downregulated genes in patients in C1 compared to C2 and C3 (**Supplementary Figure 4A**). No DEGs were observed in patients in C2 compared to C1 and C3. However, 524 upregulated and 242 downregulated genes were observed in patients in C3 compared to C1 and C2 (**Supplementary Figure 4B**). Next, we performed the “Gene ontology” and “KEGG pathway enrichment” analyses to annotate gene functions. The results revealed that these 166 upregulated genes in C1 were enriched in the protein activation pathway and high-density lipoprotein particle (**Supplementary Figure 4C**). Further, 242 downregulated genes in C3 were enriched in the small molecule catabolic processes and high-density lipoprotein particles. The “KEGG pathway enrichment” analysis showed the enrichment of the retinol metabolism, the biosynthesis of the steroid hormones, and the metabolism of xenobiotics by cytochrome P450 in C1 or C3 (**Supplementary Figure 4D**).

### 3.8 Construction of the mRNAsi model based on DEGs

**Figure 4:**
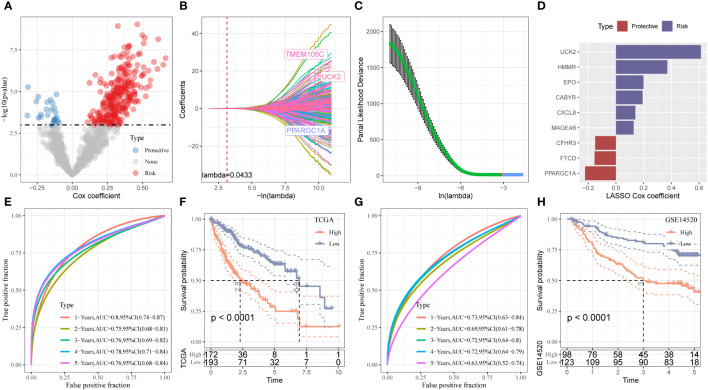
Establishment and validation of the Stemness Subtype classifier based on hub genes by machine learning methods. **(A)** Total of promising candidates were identified through the survival analysis of the mRNAsi. **(B, C)** Nine mRNAsi-related genes using the least absolute shrinkage and selection operator regression. **(D)** The coefficients for each gene in the mRNAsi-related prognostic signature. **(E)** ROC curves generated by the mRNAsi-related signature for predicting the 1/2/3/4/5-year overall survival in TCGA. **(F)** Kaplan-Meier curves showing different overall survival of patients in high- and low-risk groups based on TCGA-LIHC cohort. **(G)** ROC curves generated by the mRNAsi-related signature for predicting the 1/2/3/4/5-year overall survival in GSE14520 cohort. **(H)** Kaplan-Meier curves showing different overall survival of patients in high- and low-risk groups based on GSE14520 cohort.

We identified 816 DEGs in three stemness subtypes and performed “COX regression analysis” to identify the prognostic factors. A total of 272 risk genes and 26 protective genes associated with the OS were identified (**Figure 4A**, *P* < 0.001). We categorized patients based on the expression of mRNAsi-related DEGs and heterogeneity of stemness characteristics using unsupervised consensus clustering. The “glmnet” package was used to perform the “LASSO-COX regression” analysis. **Figure 4B** shows a gradual decrease in the regression coefficients of predictors towards zero with an increase in lambda. We determined the optimal lambda value using 10-fold cross-validation, and the results showed that the partial likelihood deviance of the model was minimized when lambda = 0.0433 (**Figure 4C**). We selected 20 genes associated with this lambda value for further analysis. The model reached the best when the lambda = 0.0433. We categorized patients into stemness subtype I (193 patients, 52.9%) and stemness subtype II (172 patients, 47.2%, **Figures 4A-C**). We calculated the riskscore as follows = 0.23 * UCK2 + 0.138 * HMMR + 0.094 * CABYR + (-0.072 * CFHR3) + (-0.148* PPARGC1A) + 0.129 * EPO + (-0.094 * FTCD) + 0.071 * CXCL8 + 0.069 * MAGEA6 (**Figure 4D**). A “time-dependent ROC” analysis was used to determine the accuracy of the model in predicting the prognosis. The AUC values of 1-, 2-, 3-, 4-, and 5-year OS were 0.8, 0.75, 0.76, 0.78, and 0.76, respectively, indicating the good predictive significance of the model (**Figure 4E**). The KM survival curve showed that the prognosis of patients in stemness subtype II was poor compared to patients in stemness subtype I (**Figure 4F**). Finally, we validated the model in GSE14520, and the results were similar (**Figures 4G, H**).

### 3.9 The performance of stemness risk score in different clinicopathological and subtypes

A significant difference in the gender, T stage, tumor stage, grade, stemness, and previous miRNAsi model was observed in patients in HRG and LRG (**Supplementary Figure 5A**). The results showed that mRNAsi was positively correlated with the degree of risk score (**Supplementary Figure 5B**). The ssGSEA method of GSVA package was used to calculate the scores of the stemness related gene sets downloaded from the molecular signature database (MSigDB) in the TCGA-LIHC cohort samples, including “WONG EMBRYONIC STEM CELL CORE”, “YAMASHITA LIVER CANCER STEM CELL UP” and “YAMASHITA LIVER CANCER STEM CELL DN”, and then correlation analysis was performed with nine-gene signature. We found that the wone embryonic stem cells core score and yamashita liver cancer stem cell up score of HCC samples were both significantly positively correlated with the risk score, respectively. While yamashita liver cancer stem cell down score was significantly negatively correlated with risk score (**Supplementary Figures 6A-C**). We also found that the high-risk group had a significantly higher wone embryonic stem cells core score and yamashita liver cancer stem cell up score, as well as a significantly lower yamashita liver cancer stem cell down score than the low-risk group (**Supplementary Figures 6D-F**). In addition, the KM survival curve showed that the prognosis of patients with high riskscores was poor compared to patients with riskscores in both mRNAsi groups (**Supplementary Figure 5C**). Moreover, the OS of patients with stage 1 and II HCC in HRG was better compared to LRG (**Supplementary Figure 5D**). A similar survival trend was observed in patients with stage III and IV HCC; however, the difference was not significant (P = 0.055). The OS of patients with grade I and II HCC in HRG was better compared to LRG. A similar survival trend was observed in patients with stage III and IV HCC (**Supplementary Figure 5E**). These results show that our stemness-based classifier could predict the patient’s prognosis and should be analyzed further.

### 3.10 Characteristics of immune and pathway between different riskscore

**Figure 5:**
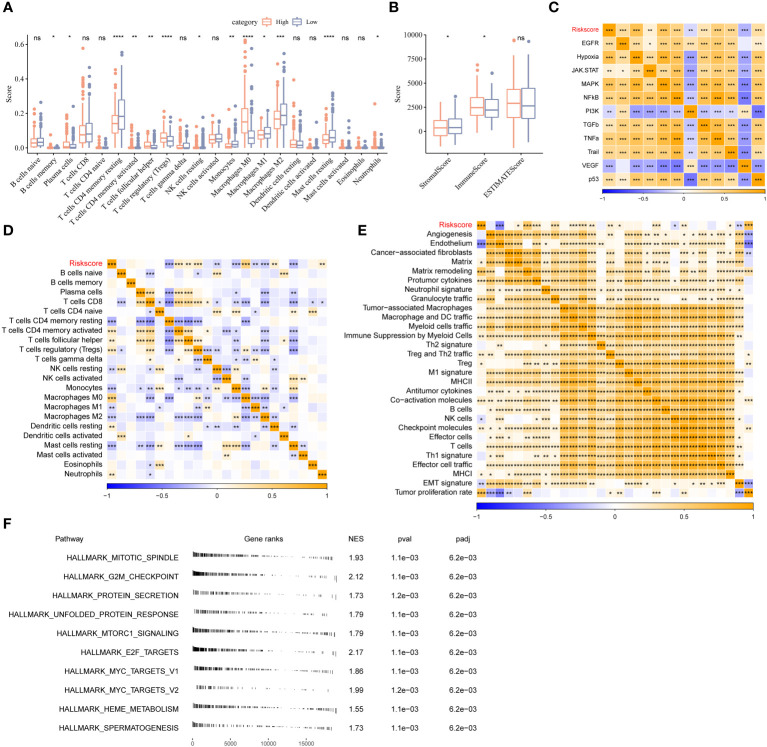
Different TIME status, oncogenic pathway, and pervious signature between two stemness subtypes. **(A)** Comparisons of the abundances of 22 immune cells in two clusters by CIBERSORT. **(B)** Comparisons of stromal score, immune score, and ESTIMATE score between two clusters. **(C)** Comparisons of the 11 oncogenic pathways in two clusters by PROGENy algorithm. **(D)** The correlation between stemness subtypes and 22 immune cells. **(E)** The correlation between the stemness subtypes and 22 TME-related signatures. **(F)** GSEA algorithm was performed with all HALLMARK gene sets in different subgroups. *P<0.05, **P<0.01, ***P<0.001, ****P<0.0001, ns, Not Significant.

To investigate the difference between the riskscore and immune characteristics, we compared the 22 immune cell types in the TIME. The infiltration of several immune cells differed in patients in HRG and LRG (**Figure 5A**). The differences in the immune cell infiltration in patients in the two stemness subtypes were analogous to the previous mRNAsi model. A difference in the abundance of macrophages was observed, wherein a significant increase in the abundance of M0 macrophages and a significant decrease in M1 and M2 macrophages in patients from TCGA-LIHC cohort in HRG (**Figure 5A**). The stromal score of patients in HRG was remarkably increased in LRG. Furthermore, the results revealed a significant increase in the immune scores of patients in HRG compared to LRG. However, no significant difference was observed in the ESTIMATE scores of patients in both riskscore groups (**Figure 5B**). Next, we analyzed the riskscore and pathway activity. The riskscore was significantly associated with the VEGF and PI3K signaling pathways, etc. (**Figure 5C**). Moreover, several immune cells were significantly associated with the riskscore (**Figure 5D**). Additionally, the riskscore was negatively associated with angiogenesis, endothelium, cancer-associated fibroblasts (CAFs), and EMT signature (**Figure 5E**). All genes from the HALLMARK database were subjected to GSEA to analyze the enrichment of the riskscores. The results revealed enrichment of the mitotic spindle, G2M checkpoint, protein secretion, unfolded protein response, and mTORC1 signaling by genes in HRG (**Figure 5F**).

### 3.11 Immunotherapy and ICB response of riskscore

**Figure 6:**
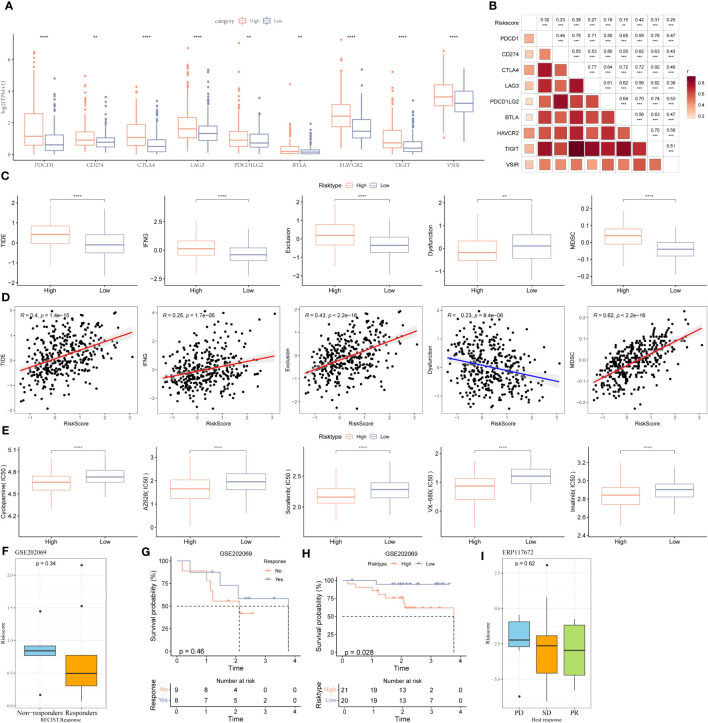
Immune landscape, immune checkpoint profile, sensitivity to targeted therapy and immunotherapy response prediction between two stemness subtypes. **(A)** Expression levels of immune checkpoints: VSIR, PDCD1LG2, CD274, HAVCR2(TIM-3), BTLA, LAG-3, TIGIT, CTLA-4, and PDCD1 (PD-1) in two clusters. **(B)** The correlation between immune checkpoints and the stemness subtypes. **(C)** Comparisons of TIDE, IFNG, T cell exclusion dysfunction, and MDSC in the high- and low-risk groups. **(D)** The correlation between TIDE score, IFNG, T cell exclusion, dysfunction, MDSC and stemness subtypes. **(E)** The boxplots of the estimated IC_50_ for cyclopamine, AZ628, sorafenib, VX-680, and imatinib in the high- and low-risk groups. **(F)** The scatter diagram showing the level of riskscore between the no-responder and responder in the GSE202069 dataset. **(G)** KM survival curve between no-responder and responder in the GSE202069 dataset. **(H)** KM survival curve between low-risk and high-risk groups in the GSE202069 dataset. **(I)** The scatter diagram showing the level of riskscore among PD, SD and PR in ERP117672 dataset. *P<0.05, **P<0.01, ***P<0.001, ns, Not Significant. **P<0.01, ***P<0.001, ****P<0.0001, ns, Not Significant.

First, we determine immune checkpoint gene expression in patients in HRG and LRG. The results showed an increase in *PDCD1, CD274, CTLA4, LAG3, PDCD1LG2, BTLA, HAVCR2, TIGHT*, and *VISIR* expression in patients in HRG (**Figure 6A**). Furthermore, there was a strong association between riskscore and immune checkpoints expression (**Figure 6B**). Next, we used the TIDE database to determine the response of patients in different riskscore groups to immunotherapy (**Figure 6C**). A significant difference was observed in the TIDE scores and MDSC abundance in patients in LRG and HRG. A significant correlation was observed between the degree of riskscore and the infiltration of MDSC, CAFs, IFNG, and the TIDE scores. Additionally, the degree of riskscore was negatively correlated with T cell dysregulation (**Figure 6D**). Cyclopamine, AZ628, sorafenib, VX-680, and imatinib are used to treat cancer patients. The results revealed that patients with high riskscore could benefit from these drugs (**Figure 6E**). Additionally, to explore the correlation between riskscore and immunotherapy, we determined the ability of riskscores to predict the patient’s response to ICB. GSE202069 dataset included a total of 41 samples, of which 17 patients received PD-1 therapy, including 9 non-responders and 8 responders. Riskscore was calculated in GSE202069 dataset using the same method as in TCGA, and the difference in riskscores between responders and non-responders was compared. The results showed that the risk score of the non-responder group was higher and the prognosis was worse (**Figures 6F, G**). The survival curve between the risk groups showed that the prognosis was worse in the high-risk group (**Figure 6H**). ERP117672 included a total of 40 RNA-seq samples from HCC patients treated with pabolizumab, including 29 non-responder samples, 6 responder samples, and 5 non-evaluable samples. The results showed that Riskscore was higher in PD and SD group than PR group (**Figure 6I**).

### 3.12 Anti-PD1 inhibits HCC progression and UCK2 expression in C57BL/6 mice

**Figure 7:**
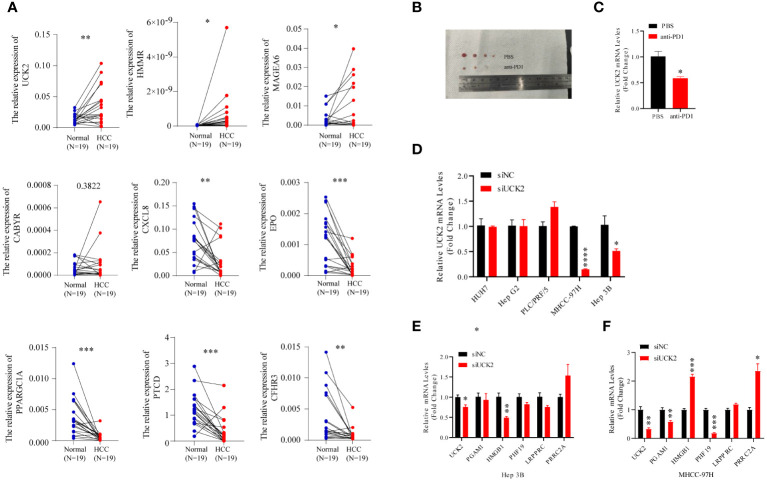
Anti-PD1 inhibits HCC progression and UCK2 expression in C57BL/6 mice. **(A)** The expression of nine genes in the clinical HCC tissues. qRT-PCR assay of nine genes mRNA expression levels in 19 pairs of HCC and adjacent tissues. **(B)** smaller size of HCC nodules was found in the mice with anti-PD1 treatment compared to the untreated mice. **(C)** Significant decreases of liver UCK2 expression was found in the anti-PD1 treated mice compared to the untreated mice. **(D)** siRNA was used to knockdown UCK2 expression in 5 HCC cell lines. **(E)** RT-qPCR results showed that siUCK2 inhibited HMGB1 expression in Hep3B cells. **(F)** Significant reduction of PGAM1, PHF19 and upregulation of HMGB1, PRRC2A in MHCC-97H cells by siUCK2. *P<0.05, **P<0.01, ***P<0.001, ****P<0.0001.

To validate the robustness of the nine gene signature, we collected tumor tissues and matched non-tumor tissues from 19 patients with HCC and determined the expression of nine genes using qRT-PCR. A significant increase in *UCK2, HMMR*, and *MAGEA6* expression and a significant decrease in *CXCL8, EPO, PPARGC1A, FTCD*, and *CFHR3* expression was observed in tumor tissues. In addition, an increase in *CABYR* expression was observed in tumor tissues; however, the increase was not statistically significant (**Figure 7A**). Because previous analyses have shown that gene signature could predict immunotherapy outcomes. Herein, we performed an anti-PD1 treatment in an HCC mice model. Macroscopically, smaller size and fewer numbers of HCC nodules were found in the mice with anti-PD1 treatment compared to the untreated mice (**Figure 7B**). Significant decreases of tumor UCK2 expression was found in the anti-PD1 treated mice compared to the untreated mice (**Figure 7C**). Furthermore, we knockdown UCK2 expression using siRNA in 5 HCC cell lines, but only effective reduction in MHCC-97H and Hep3B cells (**Figure 7D**). To understand whether UCK2 regulates the expression of immune genes, we determined immune genes expressions in MHCC-97H and Hep3B cells treated siUCK2. Those immune genes were reported to regulated HCC immune infiltration or immunotherapy (23–27). RT-qPCR results showed that siUCK2 inhibited HMGB1 expression in Hep3B cells (**Figure 7E**), and significant reduction of PGAM1, PHF19 and upregulation of HMGB1, PRRC2A in MHCC-97H cells (**Figure 7F**).

## 4 Discussion

A high level of heterogeneity is observed in CSCs; however, CSCs are not found abundantly in tumor tissues. The differentiation, indefinite proliferation, and self-renewal ability of CSCs are closely associated with the development, progression, and relapse of tumor and drug resistance (28). Earlier studies on HCC mostly focused on a single gene, ignoring the importance of multi-gene combination in predicting HCC (29, 30). Chemoresistance and increased incidences of cancer relapse significantly increase the mortalities of patients with HCC. Recent studies have used existing CSC markers and algorithms to calculate a comprehensive mRNAsi. In this study, we determined the comprehensive features of HCC stemness by analyzing gene expression patterns in two cohorts using bioinformatic techniques. First, we employed the OCLR machine-learning algorithm to calculate the mRNAsi of all patients with HCC. Next, we used the consensus clustering method and identified three distinct stemness clusters to predict the patient prognosis. The AUC values for different OS years of patients in TCGA-LIHC cohort was > 0.75, thereby indicating the robustness of the mRNAsi model for predicting the patient prognosis. We also validated the model in patients in GSE14520. Next, we evaluated the differences between the three subgroups in clinicopathological features, the signaling pathway, immune cell infiltration, and immune profiles of patients with HCC. Furthermore, we constructed a mRNAsi model based on the DEGs in these three stemness subtypes, which could effectively predict the patient’s survival and was closely linked to the TME of patients with HCC. In addition, the mRNAsi model was associated with immune-related pathways and could predict the response of patients to immune and chemotherapies.

We screened nine genes from 298 mRNAsi-related DEGs with prognostic value in patients in three stemness clusters using the “LASSO” algorithm to construct a clinically applicable predictor for the stemness subtype. Of these nine genes, *UCK2, HMMR, EPO, CABYR, CXCL8*, and *MAGEA6* were risk genes, and *CFHR3, FTCD*, and *PARGC1A* were protective genes. Studies have shown an increase in *UCK2* (31), *HMMR* (32), *CABYR* (33), and *MAGEA6* (34) expression, and a significant decrease in *CFHR3* (35), *FTCD* (36), and *PARGC1A* (37) expression in patients with HCC. Consistent with previous studies, these genes could promote the development and metastasis of cancer cells, thereby leading to unfavorable outcomes in patients in HRG. Of these nine genes, a study has shown the involvement of *UCK2* (38) in cancer stemness. *HMMR* is involved in cellular adipogenesis, HCC, and nonalcoholic steatohepatitis (39). EPO receptors are expressed by cancer cells, which increases the suppression of macrophages-regulated T cells suppression (40, 41). The role of CXCL8 in the tumor microenvironment (TME) is complex. Our results showed a decrease in *CXCL8* expression in HCC tissues; however, the bioinformatic analysis showed an increase in *CXCL8* expression in patients with HCC, associated with poor prognosis. These discrepancies could arise due to different sources and downstream effects of *CXCL8* in HCC tumor and non-tumor tissues. Monocytes derived-CXCL8 recruit neutrophils to promote a metastatic TME in HCC (42). CXCL8 induced by the hepatitis B virus promotes Treg cell accumulation in the liver (43). A cuproptosis-related prognostic signature consisting of *MAGEA6* and *EPO* promotes HCC development (44). A correlation was observed between *CFHR3* and hypoxia (45). The level of FTCD served as a powerful diagnostic predictor for distinguishing early-stage HCC from benign tumors (36). *PPARGC1A*, also known as PGC-α, regulates tumor metabolism and suppresses the metastasis of HCC cells by inhibiting the Warburg effect (46). Subsequently, we explored the probability of the stemness signature in clinical application. We evaluated the correlation between clinical features and riskscores of patients, and the results revealed a close association between stemness signature, T and N stages, and tumor stage and grade. Our model could predict the survival of patients with different stages or grades of HCC.

Immunotherapies have revolutionized the treatment of patients with advanced cancers. ICB targeting PD-1, PD-L1, and CTLA-4 have made significant progress in treating patients with HCC. However, the objective response rate of ICBs as monotherapy in treating patients with HCC is only 15%–20% (47). Since CSCs play a crucial role in the metastasis and recurrence of cancers, targeting CSCs could provide long-term benefits in cancer treatment (48). Therefore, identifying CSC-related biomarkers for HCC could enhance our understanding of the mechanism of stemness and aid in discovering novel stemness-related therapeutic targets. We constructed a mRNAsi model based on nine CSCs-related genes and validated the performance of the model in GSE14520. This gene signature was closely associated with HCC prognosis, providing valuable guidance for developing prognostic factors related to HCC stemness. We identified *HMMR, EPO, CABYR, CXCL8*, and *MAGEA6* as novel CSC-related genes. Previous studies have an association between these genes and stemness in TME of HCC. Immune or stromal cells can modify CSC functions, thereby affecting the behavior of cancer cells. The TME maintains the stemness features of CSCs and aids in transforming cancer cells into CSCs, thereby impacting therapeutic efficacy (49). Studies have shown that CLCF1 produced by CAFs can stimulate TGF-β1 and CXCL6 secretion by cancer cells, thereby increasing the stemness of cancer cells and activating the ERK1/2 signaling pathway in CAFs. This induces the production of CLCF1, thereby creating a positive feedback loop to promote HCC progression (50). *In vitro* studies have revealed that lymphatic endothelial cells secrete IL-17A and closely interact with CD133^+^ HCC cells to promote self-renewal and tumorigenesis of hepatic stem cells. Additionally, CD133^+^ CSCs stimulate IL-17A expression in lymphatic endothelial cells (51). Tumor-associated neutrophils expressing CCL2+ or CCL17+ promote BMP-2 and TGF-β2 secretion, thereby enhancing miR-301b-3p expression via paracrine signaling and activating the NF-κB signaling pathway. Together, this axis promotes HCC stemness. Additionally, these HCC cells increase CXCL5 expression, thereby increasing neutrophil infiltration in TME (52). Moreover, the underlying mechanism of HMMR, EPO, CABYR, CXCL8, and MAGEA6 in HCC stemness requires additional investigation.

Despite the advancement in the field of chemo and immunotherapies, the therapeutic outcomes of patients with advanced HCC are still unsatisfactory due to the resistance of CSCs to conventional therapeutic options. CSCs are resistant to standard therapy; hence, there is an urgent need to design innovative therapeutic approaches specifically targeting CSCs. Stemness signatures and HCC targeting compounds were identified using the pRRophetic database. Except for sorafenib, the Hedgehog signaling pathway inhibitors, such as cyclopamine, AZ628, Raf inhibitor, VX-680, aurora kinase inhibitor, and imatinib, a tyrosine kinase inhibitor, are not used for treating patients with the HCC, thereby providing new opportunities for discovering drugs for HCC treatment. CSCs regulate immunotherapy responses; hence, combining traditional and multimodal therapy targeting CSCs and TME could effectively eliminate cancer cells.

Our research has shown a close correlation between CSCs and the immune microenvironment status of tumors. We developed the mRNAsi (Cluster I, II, and III) and the stemness subtype (Subtype I vs. Subtype II) models. The results revealed that patients with more prominent stemness characteristics or patients with poor prognoses had immunosuppressive TME, high immune checkpoint gene expression, and low stromal scores. These results indicate that our models could be used for patient follow-up and has clinical applications. Additionally, our models could predict the immune response to tumors and the response of patients to immunotherapy. Our results showed that patients with high-risk scores were more sensitive to ICIs. Interestingly, a significant difference in TMBs and the exclusion and dysfunction of T cells in patients in HRG and LRG. This correlation between the risk score and ICI response could be due to the positive correlation between the risk score and immune checkpoint gene expressions, such as *PD-1, HAVCR2, LAG3*, and *CTLA4*.

## 5 Conclusion

In summary, our study provides evidence that CSCs regulate immune cell infiltration and determine the response of patients with HCC to immunotherapy. In addition, we have developed a novel and practical HCC stemness subtype classifier, which could be used to investigate the relationship between CSCs and TME and identify individuals who could benefit from immunotherapy.

## Data availability statement

The datasets presented in this study can be found in online repositories. The names of the repository/repositories and accession number(s) can be found in the article/**Supplementary Material**. All date and materials are available from the corresponding authors upon request.

## Ethics statement

The studies involving humans were approved by the ethics committee of Peking University Shenzhen Hospital. The studies were conducted in accordance with the local legislation and institutional requirements. The participants provided their written informed consent to participate in this study. The animal study was approved by the Ethics Committee of Peking University Shenzhen Hospital. The study was conducted in accordance with the local legislation and institutional requirements.

## Author contributions

EC designed the study. EC, ZZ, and RW obtained the data from database. EC and ZZ performed analysis of the data. EC and ZZ wrote the manuscript. RW, JL, ZP, and ZG performed experimental analysis. EC, JKL, and ZL revised the manuscript. All authors contributed to the article and approved the submitted version.

## Supplementary material

Supplementary Figure 1 The relationship between the clinical features and mRNAsi scores in HCC patients. **(A)** The Kaplan-Meier plot displayed a worse prognosis of patients showing a high level of miRNAsi. **(B)** An overview of the relationship between the clinicalpathological features of HCC patients and mRNAsi. **(C)** The mRNAsi scores calculated in terms of LUAD tumor stages in TCGA-LIHC dataset. **(D, E)** The Kaplan-Meier plot displayed a worse prognosis in patients at the **(D)** stage I+II and **(E)** stage III+IV with a high level of miRNAsi. **(F)** An overview of the relationship between stage I-IV biomarkers of HCC and mRNAsi. **(F)** The mRNAsi scores calculated in terms of LUAD tumor grade in the TCGA-LIHC dataset. **(G, H)** The Kaplan-Meier plot displayed a worse prognosis of patients in **(G)** G1+G2 and **(H)** G3+G4 stage with a high level of miRNAsi. *P<0.05, **P<0.01, ns, Not Significant.

Supplementary Figure 2 Various clinicalpathological features between two stemness subtypes/An overview of the relationship between clinicalpathological features of HCC patients and mRNAsi. Patients with **(A)** younger age, **(B)** later T stage, **(C)** advanced TNM stage, **(D)** later tumor grade, and **(E)** mRNAsi score tended to show higher risk scores.

Supplementary Figure 3 The two stemness subtypes showed distinctly different functional annotations, genetic profiles, and tumor mutation status **(A-E)** The correlation between **(A)** Aneuploidy Score, **(B)** Homologous Recombination Defects, **(C)** Fraction Altered, **(D)** Number of Segments and **(E)** Tumor mutation burden. **(F)** An overview of the correlation between previously classified molecular subtype of HCC patients and mRNAsi. **(G)** The mutation frequency of the 10 most frequently mutated genes in the three clusters was shown in waterfall plot. *P<0.05, **P<0.01, ***P<0.001, ****P<0.0001, ns, Not Significant.

Supplementary Figure 4 DEGs were subjected to GO and KEGG functional enrichment analyses. **(A)** DEGs related to mRNAsi between C1 vs C2+C3 were screened. **(B)** DEGs related to mRNAsi between C3 vs C1+C2 were screened. **(C)** Overexpressed DEGs from C1 vs other were subjected to GO and KEGG functional enrichment analyses. **(D)** Down-regulated DEGs from C3 vs other were subjected to GO and KEGG functional enrichment analyses.

Supplementary Figure 5 Correlation between clinicopathological features and mRNAsi-related model. **(A)** An overview of the relationship between clinicalpathological features of HCC and riskscore. **(B)** In the TCGA-LIHC cohort, the relationship between riskscore and mRNAsi was analyzed. **(C-E)** The high-risk group in the TCGA-LIHC cohort had a poor prognosis among different stratified subgroups. ****P<0.0001.

Supplementary Figure 6 The correlation between stemness-related gene sets and mRNAsi model. **(A-C)** Correlation analysis between the riskscore and wone embryonic stem cells core score and yamashita liver cancer stem cell up score, respectively. **(D-F)** The differences between high- and low- group in terms of wone embryonic stem cells core score, yamashita liver cancer stem cell up score, and yamashita liver cancer stem cell up score.

## References

[1] Prager BC Xie Q Bao S Rich JN. Cancer stem cells: the architects of the tumor ecosystem. Cell Stem Cell. (2019) 24:41–53. doi: 10.1016/j.stem.2018.12.009 30609398 PMC6350931

[2] Nassar D Blanpain C. Cancer stem cells: basic concepts and therapeutic implications. Annu Rev Pathol. (2016) 11:47–76. doi: 10.1146/annurev-pathol-012615-044438 27193450

[3] Tsui YM Chan LK Ng IO. Cancer stemness in hepatocellular carcinoma: mechanisms and translational potential. Br J Cancer. (2020) 122:1428–40. doi: 10.1038/s41416-020-0823-9 PMC7217836 32231294

[4] Pan S Zhan Y Chen X Wu B Liu B. Identification of biomarkers for controlling cancer stem cell characteristics in bladder cancer by network analysis of transcriptome data stemness indices. Front Oncol. (2019) 9:613. doi: 10.3389/fonc.2019.00613 31334127 PMC6620567

[5] Malta TM Sokolov A Gentles AJ Burzykowski T Poisson L Weinstein JN. Machine learning identifies stemness features associated with oncogenic dedifferentiation. Cell. (2018) 173:338–54.e15. doi: 10.1016/j.cell.2018.03.034 29625051 PMC5902191

[6] Shu X Wang Q Wu Q. The eph/ephrin system in hepatocellular carcinoma: functional roles and potential therapeutic targets. Oncologie. (2022) 24:427–39. doi: 10.32604/oncologie.2022.023248

[7] Finn RS Qin S Ikeda M Galle PR Ducreux M Kim TY. Atezolizumab plus bevacizumab in unresectable hepatocellular carcinoma. N Engl J Med. (2020) 382:1894–905. doi: 10.1056/NEJMoa1915745 32402160

[8] Sangro B Sarobe P Hervas-Stubbs S Melero I. Advances in immunotherapy for hepatocellular carcinoma. Nat Rev Gastroenterol Hepatol. (2021) 18:525–43. doi: 10.1038/s41575-021-00438-0 PMC8042636 33850328

[9] Yan D Li C Zhou Y Yan X Zhi W Qian H. Exploration of combinational therapeutic strategies for HCC based on TCGA HCC database. Oncologie. (2022) 24:101–11. doi: 10.32604/oncologie.2022.020357

[10] Ritchie ME Phipson B Wu D Hu Y Law CW Shi W. limma powers differential expression analyses for RNA-sequencing and microarray studies. Nucleic Acids Res. (2015) 43:e47. doi: 10.1093/nar/gkv007 25605792 PMC4402510

[11] Wilkerson MD Hayes DN. ConsensusClusterPlus: a class discovery tool with confidence assessments and item tracking. Bioinformatics. (2010) 26:1572–3. doi: 10.1093/bioinformatics/btq170 PMC2881355 20427518

[12] Newman AM Liu CL Green MR Gentles AJ Feng W Xu Y. Robust enumeration of cell subsets from tissue expression profiles. Nat Methods. (2015) 12:453–7. doi: 10.1038/nmeth.3337 PMC4739640 25822800

[13] Yoshihara K Shahmoradgoli M Martinez E Vegesna R Kim H Torres-Garcia W. Inferring tumour purity and stromal and immune cell admixture from expression data. Nat Commun. (2013) 4:2612. doi: 10.1038/ncomms3612 24113773 PMC3826632

[14] Liberzon A Birger C Thorvaldsdottir H Ghandi M Mesirov JP Tamayo P. The Molecular Signatures Database (MSigDB) hallmark gene set collection. Cell Syst. (2015) 1:417–25. doi: 10.1016/j.cels.2015.12.004 PMC4707969 26771021

[15] Barretina J Caponigro G Stransky N Venkatesan K Margolin AA Kim S. The Cancer Cell Line Encyclopedia enables predictive modelling of anticancer drug sensitivity. Nature. (2012) 483:603–7. doi: 10.1038/nature11003 PMC3320027 22460905

[16] Geeleher P Cox N Huang RS. pRRophetic: an R package for prediction of clinical chemotherapeutic response from tumor gene expression levels. PLoS One. (2014) 9:e107468. doi: 10.1371/journal.pone.0107468 25229481 PMC4167990

[17] Chen E He Y Jiang J Yi J Zou Z Song Q. CDCA8 induced by NF-YA promotes hepatocellular carcinoma progression by regulating the MEK/ERK pathway. Exp Hematol Oncol. (2023) 12:9. doi: 10.1186/s40164-022-00366-y 36639822 PMC9838039

[18] Davoli T Uno H Wooten EC Elledge SJ. Tumor aneuploidy correlates with markers of immune evasion and with reduced response to immunotherapy. Science. (2017) 355. doi: 10.1126/science.aaf8399 PMC5592794 28104840

[19] Thorsson V Gibbs DL Brown SD Wolf D Bortone DS Ou Yang TH. The immune landscape of cancer. Immunity. (2018) 48:812–30.e14. doi: 10.1016/j.immuni.2018.03.023 29628290 PMC5982584

[20] Bagaev A Kotlov N Nomie K Svekolkin V Gafurov A Isaeva O. Conserved pan-cancer microenvironment subtypes predict response to immunotherapy. Cancer Cell. (2021) 39:845–65.e7. doi: 10.1016/j.ccell.2021.04.014 34019806

[21] Schubert M Klinger B Klunemann M Sieber A Uhlitz F Sauer S. Perturbation-response genes reveal signaling footprints in cancer gene expression. Nat Commun. (2018) 9:20. doi: 10.1038/s41467-017-02391-6 29295995 PMC5750219

[22] Hu J Yu A Othmane B Qiu D Li H Li C. Siglec15 shapes a non-inflamed tumor microenvironment and predicts the molecular subtype in bladder cancer. Theranostics. (2021) 11:3089–108. doi: 10.7150/thno.53649 PMC7847675 33537076

[23] Zheng Y Wang Y Lu Z Wan J Jiang L Song D. PGAM1 inhibition promotes HCC ferroptosis and synergizes with anti-PD-1 immunotherapy. Adv Sci (Weinh). (2023) 10:e2301928. doi: 10.1002/advs.202301928 37705495 PMC10582428

[24] Ye L Zhang Q Cheng Y Chen X Wang G Shi M. Tumor-derived exosomal HMGB1 fosters hepatocellular carcinoma immune evasion by promoting TIM-1(+) regulatory B cell expansion. J Immunother Cancer. (2018) 6:145. doi: 10.1186/s40425-018-0451-6 30526680 PMC6288912

[25] Zhu ZY Tang N Wang MF Zhou JC Wang JL Ren HZ. Comprehensive pan-cancer genomic analysis reveals PHF19 as a carcinogenic indicator related to immune infiltration and prognosis of hepatocellular carcinoma. Front Immunol. (2021) 12:781087. doi: 10.3389/fimmu.2021.781087 35069553 PMC8766761

[26] Wang H Tang A Cui Y Gong H Li H. LRPPRC facilitates tumor progression and immune evasion through upregulation of m(6)A modification of PD-L1 mRNA in hepatocellular carcinoma. Front Immunol. (2023) 14:1144774. doi: 10.3389/fimmu.2023.1144774 37063837 PMC10097877

[27] Liu X Zhang Y Wang Z Liu L Zhang G Li J. PRRC2A promotes hepatocellular carcinoma progression and associates with immune infiltration. J Hepatocell Carcinoma. (2021) 8:1495–511. doi: 10.2147/JHC.S337111 PMC8646232 34881207

[28] Lee TK Guan XY Ma S. Cancer stem cells in hepatocellular carcinoma - from origin to clinical implications. Nat Rev Gastroenterol Hepatol. (2022) 19:26–44. doi: 10.1038/s41575-021-00508-3 34504325

[29] Yu S Lv H Zhang J Zhang H Ju W Jiang Y. Heparanase/syndecan-1 axis regulates the grade of liver cancer and proliferative ability of hepatocellular carcinoma cells. Oncologie. (2022) 24:539–51. doi: 10.32604/oncologie.2022.024882

[30] Wang L Ma X Chen Y Zhang J Zhang J Wang W. MiR-145-5p suppresses hepatocellular carcinoma progression by targeting ABHD17C. Oncologie. (2022) 24:897–912. doi: 10.32604/oncologie.2022.025693

[31] Wang Z Fu Y Xia A Chen C Qu J Xu G. Prognostic and predictive role of a metabolic rate-limiting enzyme signature in hepatocellular carcinoma. Cell Prolif. (2021) 54:e13117. doi: 10.1111/cpr.13117 34423480 PMC8488553

[32] Yuan C Yuan M Chen M Ouyang J Tan W Dai F. Prognostic implication of a novel metabolism-related gene signature in hepatocellular carcinoma. Front Oncol. (2021) 11:666199. doi: 10.3389/fonc.2021.666199 34150630 PMC8213025

[33] Yu XN Guo HY Liu TT Zhang GC Zhu HR Shi X. Upregulated calcium-binding tyrosine phosphorylation-regulated protein-a/b regulates cell proliferation and apoptosis and predicts poor prognosis in hepatocellular carcinoma. J Cell Biochem. (2020) 121:2938–49. doi: 10.1002/jcb.29538 31692072

[34] Song ZB Yu Y Zhang GP Li SQ. Genomic instability of mutation-derived gene prognostic signatures for hepatocellular carcinoma. Front Cell Dev Biol. (2021) 9:728574. doi: 10.3389/fcell.2021.728574 34676211 PMC8523793

[35] Wan Z Li X Luo X Wang B Zhou X Chen A. The miR-590-3p/CFHR3/STAT3 signaling pathway promotes cell proliferation and metastasis in hepatocellular carcinoma. Aging (Albany NY). (2022) 14:5783–99. doi: 10.18632/aging.204178 PMC9365569 35852862

[36] Seimiya M Tomonaga T Matsushita K Sunaga M Oh-Ishi M Kodera Y. Identification of novel immunohistochemical tumor markers for primary hepatocellular carcinoma; clathrin heavy chain and formiminotransferase cyclodeaminase. Hepatology. (2008) 48:519–30. doi: 10.1002/hep.v48:2 18571811

[37] Zhou S Li M Ostrow D Ruble D Mascarenhas L Pawel B. Potential methylation-regulated genes and pathways in hepatocellular neoplasm, not otherwise specified. Front Oncol. (2022) 12:952325. doi: 10.3389/fonc.2022.952325 36212481 PMC9532972

[38] Wu D Zhang C Liao G Leng K Dong B Yu Y. Targeting uridine-cytidine kinase 2 induced cell cycle arrest through dual mechanism and could improve the immune response of hepatocellular carcinoma. Cell Mol Biol Lett. (2022) 27:105. doi: 10.1186/s11658-022-00403-y 36447138 PMC9707060

[39] Zhang D Liu J Xie T Jiang Q Ding L Zhu J. Oleate acid-stimulated HMMR expression by CEBPalpha is associated with nonalcoholic steatohepatitis and hepatocellular carcinoma. Int J Biol Sci. (2020) 16:2812–27. doi: 10.7150/ijbs.49785 PMC7545721 33061798

[40] Wood MA Goldman N DePierri K Somerville J Riggs JE. Erythropoietin increases macrophage-mediated T cell suppression. Cell Immunol. (2016) 306-307:17–24. doi: 10.1016/j.cellimm.2016.05.004 27262376 PMC4983461

[41] Wang T Dai L Shen S Yang Y Yang M Yang X. Comprehensive molecular analyses of a macrophage-related gene signature with regard to prognosis, immune features, and biomarkers for immunotherapy in hepatocellular carcinoma based on WGCNA and the LASSO algorithm. Front Immunol. (2022) 13:843408. doi: 10.3389/fimmu.2022.843408 35693827 PMC9186446

[42] Peng ZP Jiang ZZ Guo HF Zhou MM Huang YF Ning WR. Glycolytic activation of monocytes regulates the accumulation and function of neutrophils in human hepatocellular carcinoma. J Hepatol. (2020) 73:906–17. doi: 10.1016/j.jhep.2020.05.004 32407813

[43] Zhang C Gao Y Du C Markowitz GJ Fu J Zhang Z. Hepatitis B-induced IL8 promotes hepatocellular carcinoma venous metastasis and intrahepatic Treg accumulation. Cancer Res. (2021) 81:2386–98. doi: 10.1158/0008-5472.CAN-20-3453 33653774

[44] Wang XX Wu LH Ji H Liu QQ Deng SZ Dou QY. A novel cuproptosis-related prognostic signature and potential value in HCC immunotherapy. Front Mol Biosci. (2022) 9:1001788. doi: 10.3389/fmolb.2022.1001788 36250008 PMC9556951

[45] Ren P Wang JY Zeng ZR Li NX Chen HL Peng XG. A novel hypoxia-driven gene signature that can predict the prognosis and drug resistance of gliomas. Front Genet. (2022) 13:976356. doi: 10.3389/fgene.2022.976356 36118887 PMC9478203

[46] Zuo Q He J Zhang S Wang H Jin G Jin H. PPARgamma coactivator-1alpha suppresses metastasis of hepatocellular carcinoma by inhibiting Warburg effect by PPARgamma-dependent WNT/beta-catenin/pyruvate dehydrogenase kinase isozyme 1 axis. Hepatology. (2021) 73:644–60. doi: 10.1002/hep.31280 32298475

[47] Flynn MJ Sayed AA Sharma R Siddique A Pinato DJ. Challenges and opportunities in the clinical development of immune checkpoint inhibitors for hepatocellular carcinoma. Hepatology. (2019) 69:2258–70. doi: 10.1002/hep.30337 30382576

[48] Pan Q Li Q Liu S Ning N Zhang X Xu Y. Concise review: targeting cancer stem cells using immunologic approaches. Stem Cells. (2015) 33:2085–92. doi: 10.1002/stem.2039 PMC4478204 25873269

[49] Wu B Shi X Jiang M Liu H. Cross-talk between cancer stem cells and immune cells: potential therapeutic targets in the tumor immune microenvironment. Mol Cancer. (2023) 22:38. doi: 10.1186/s12943-023-01748-4 36810098 PMC9942413

[50] Song M He J Pan QZ Yang J Zhao J Zhang YJ. Cancer-associated fibroblast-mediated cellular crosstalk supports hepatocellular carcinoma progression. Hepatology. (2021) 73:1717–35. doi: 10.1002/hep.31792 33682185

[51] Wei Y Shi D Liang Z Liu Y Li Y Xing Y. IL-17A secreted from lymphatic endothelial cells promotes tumorigenesis by upregulation of PD-L1 in hepatoma stem cells. J Hepatol. (2019) 71:1206–15. doi: 10.1016/j.jhep.2019.08.034 31499129

[52] Zhou SL Zhou ZJ Hu ZQ Huang XW Wang Z Chen EB. Tumor-associated neutrophils recruit macrophages and T-regulatory cells to promote progression of hepatocellular carcinoma and resistance to sorafenib. Gastroenterology. (2016) 150:1646–58.e17. doi: 10.1053/j.gastro.2016.02.040 26924089

