# Supplementary material for: Predictive value of a stemness-based classifier for prognosis and immunotherapy response of hepatocellular carcinoma based on bioinformatics and machine-learning strategies
Source: Front Immunol. 2024 Apr 17;15:1244392. doi: 10.3389/fimmu.2024.1244392 (PMC11061862; doi:10.3389/fimmu.2024.1244392)
Supplement: Supplementary file 1 [file Table_1.docx]

**Supplementary Table 1. Primer sequences of genes**

| **Genes** | **Forward (5’-3’)** | **Reverse (5’-3’)** |
| --- | --- | --- |
| PGAM1 | GTGCAGAAGAGAGCGATCCG | CGGTTAGACCCCCATAGTGC |
| HMGB1 | ACATCCAAAATCTTGATCAGTTA | AGGACAGACTTTCAAAATGTTT |
| PHF19 | ACTCGGGACTCCTATGGTGC | CCTCCGTCAGTTTGGACATCA |
| LRPPRC | GCTCATAGGATATGGGACACACT | CCAGGAAATCAGTTGGTGAGAAT |
| PRRC2A | TGGCAGCAGCAGTGGAGGAG | TGGAGGACGACTTCGGTTCTTGG |
| UCK2 | GCCCTTCCTTATAGGCGTCAG | CTTCTGGCGATAGTCCACCTC |
| HMMR | AGAACCAACTCAAGCAACAGG | AGGAGACGCCACTTGTTAATTTC |
| MAGEA6 | CGGTCACAAAGGCAGAAAT | AGGCAGGTGGCAAAGATG |
| CABYR | TGAGCAAACGGAAGCAGTTG | ACTAACTCGTGGTGACTGCT |
| CXCL8 | ACTGAGAGTGATTGAGAGTGGAC | AACCCTCTGCACCCAGTTTTC |
| EPO | GGGGGTGCCCGAACG | GGCCCCCAGAATATCACTGC |
| PPARGC1A | TCTGAGTCTGTATGGAGTGACAT | CCAAGTCGTTCACATCTAGTTCA |
| CFHR3 | TGGGCATTAGTCAAGAATACAGTAAAA | ATTAATGCCGCTTCAATATGACTTT |
| β-actin | CTTAGTTGCGTTACACCCTTTCTTG | CTGTCACCTTCACCGTTCCAGTTT |
