# Supplementary figures and images for: Predictive value of a stemness-based classifier for prognosis and immunotherapy response of hepatocellular carcinoma based on bioinformatics and machine-learning strategies
Source: Front Immunol. 2024 Apr 17;15:1244392. doi: 10.3389/fimmu.2024.1244392 (PMC11061862; doi:10.3389/fimmu.2024.1244392)

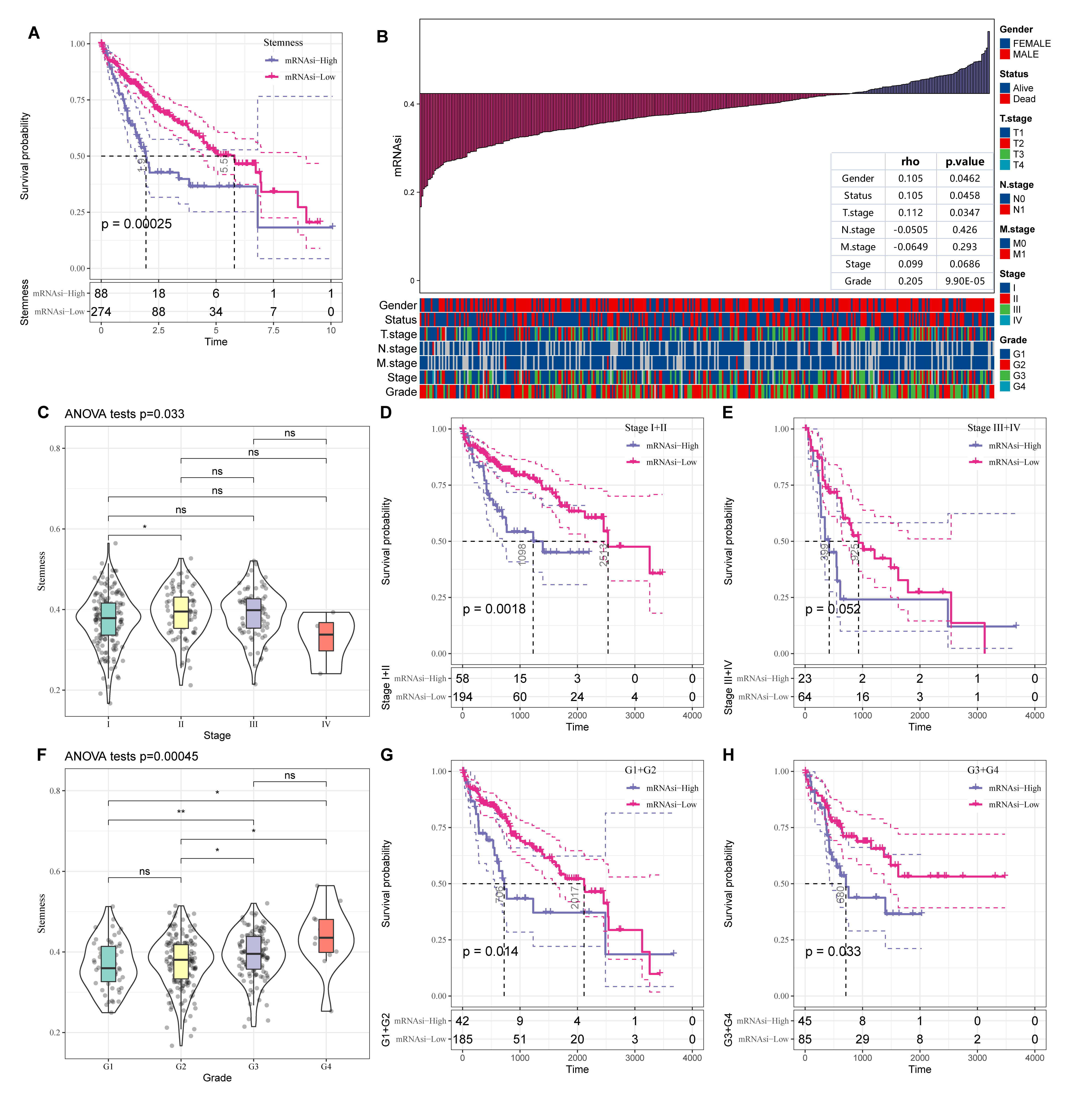

Supplement: Supplementary Figure 1 — The relationship between the clinical features and mRNAsi scores in HCC patients. (A) The Kaplan-Meier plot displayed a worse prognosis of patients showing a high level of miRNAsi. (B) An overview of the relationship between the clinicalpathological features of HCC patients and mRNAsi. (C) The mRNAsi scores calculated in terms of LUAD tumor stages in TCGA-LIHC dataset. (D, E) The Kaplan-Meier plot displayed a worse prognosis in patients at the (D) stage I+II and (E) stage III+IV with a high level of miRNAsi. (F) An overview of the relationship between stage I-IV biomarkers of HCC and mRNAsi. (F) The mRNAsi scores calculated in terms of LUAD tumor grade in the TCGA-LIHC dataset. (G, H) The Kaplan-Meier plot displayed a worse prognosis of patients in (G) G1+G2 and (H) G3+G4 stage with a high level of miRNAsi. *P<0.05, **P<0.01, ns, Not Significant. [file Image_1.tif]

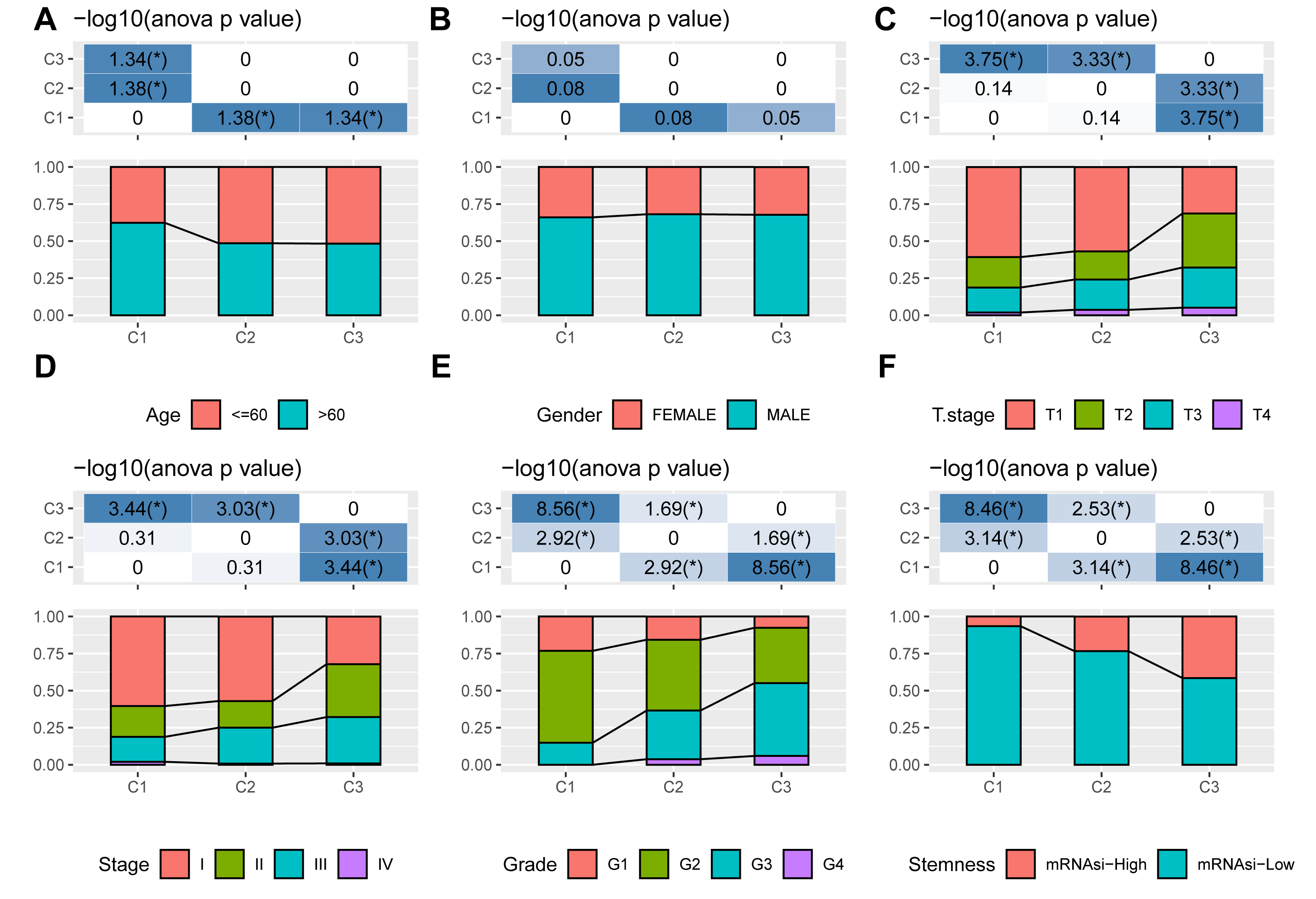

Supplement: Supplementary Figure 2 — Various clinicalpathological features between two stemness subtypes/An overview of the relationship between clinicalpathological features of HCC patients and mRNAsi. Patients with (A) younger age, (B) later T stage, (C) advanced TNM stage, (D) later tumor grade, and (E) mRNAsi score tended to show higher risk scores. [file Image_2.tif]

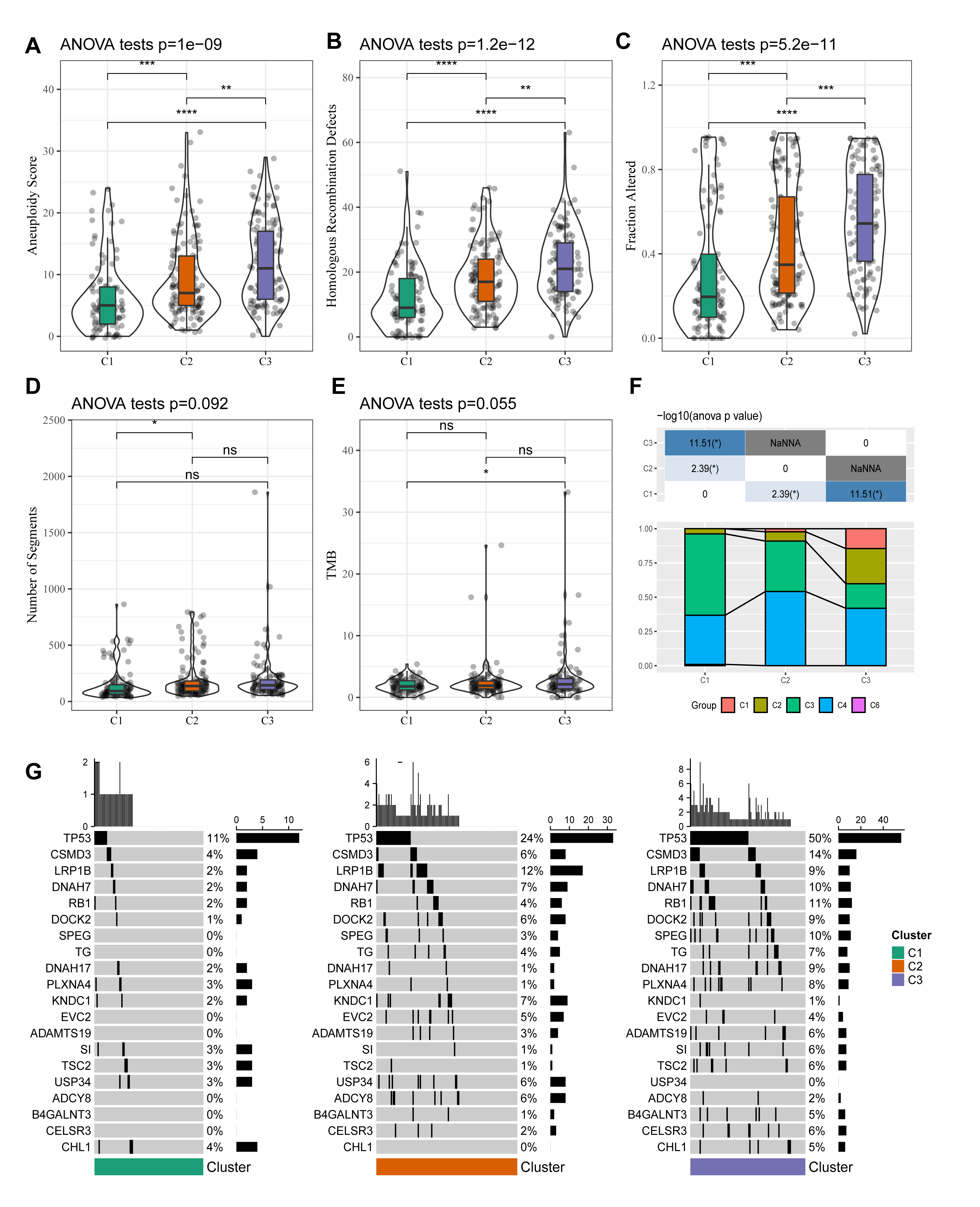

Supplement: Supplementary Figure 3 — The two stemness subtypes showed distinctly different functional annotations, genetic profiles, and tumor mutation status (A-E) The correlation between (A) Aneuploidy Score, (B) Homologous Recombination Defects, (C) Fraction Altered, (D) Number of Segments and (E) Tumor mutation burden. (F) An overview of the correlation between previously classified molecular subtype of HCC patients and mRNAsi. (G) The mutation frequency of the 10 most frequently mutated genes in the three clusters was shown in waterfall plot. *P<0.05, **P<0.01, ***P<0.001, ****P<0.0001, ns, Not Significant. [file Image_3.tif]

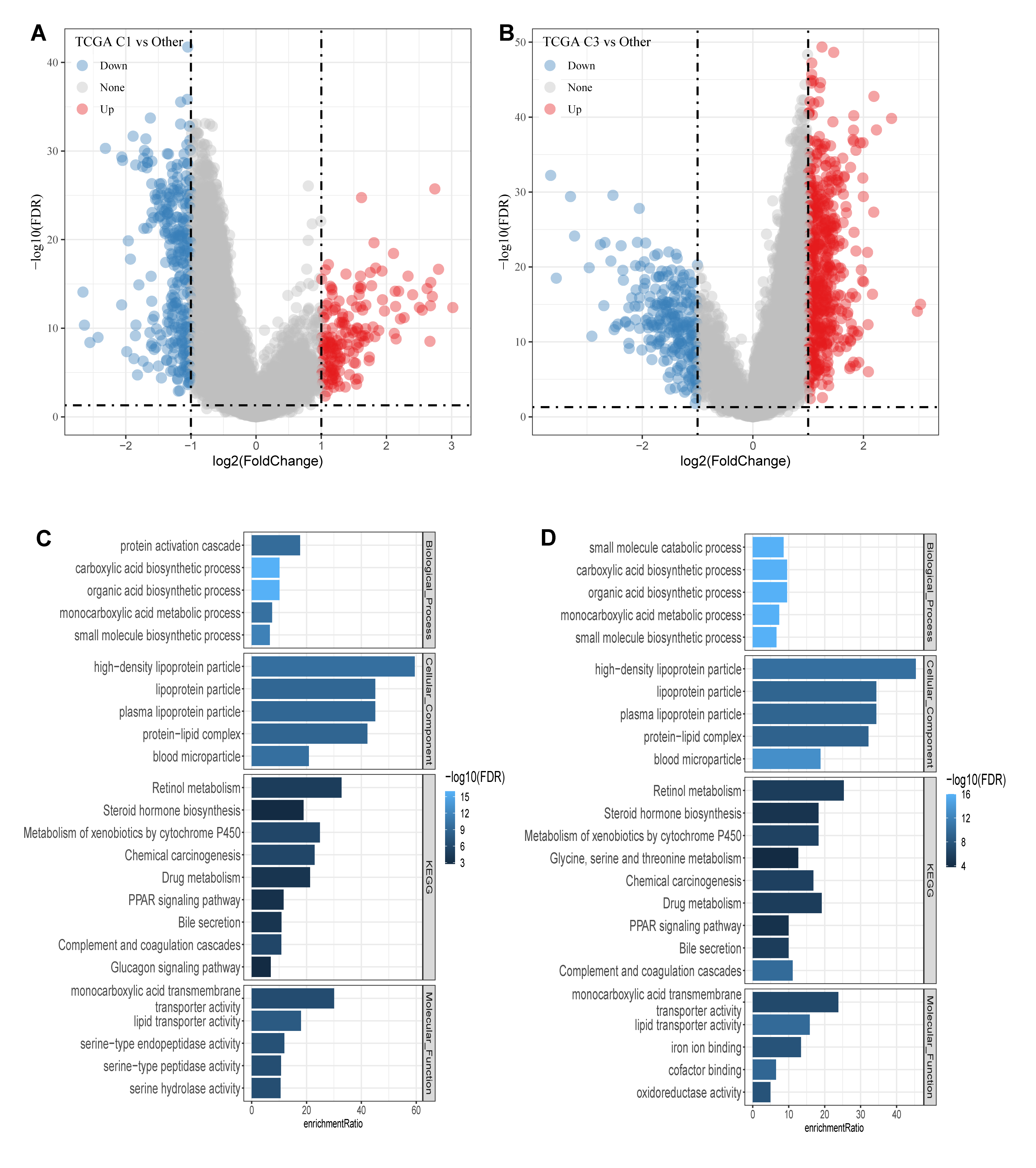

Supplement: Supplementary Figure 4 — DEGs were subjected to GO and KEGG functional enrichment analyses. (A) DEGs related to mRNAsi between C1 vs C2+C3 were screened. (B) DEGs related to mRNAsi between C3 vs C1+C2 were screened. (C) Overexpressed DEGs from C1 vs other were subjected to GO and KEGG functional enrichment analyses. (D) Down-regulated DEGs from C3 vs other were subjected to GO and KEGG functional enrichment analyses. [file Image_4.tif]

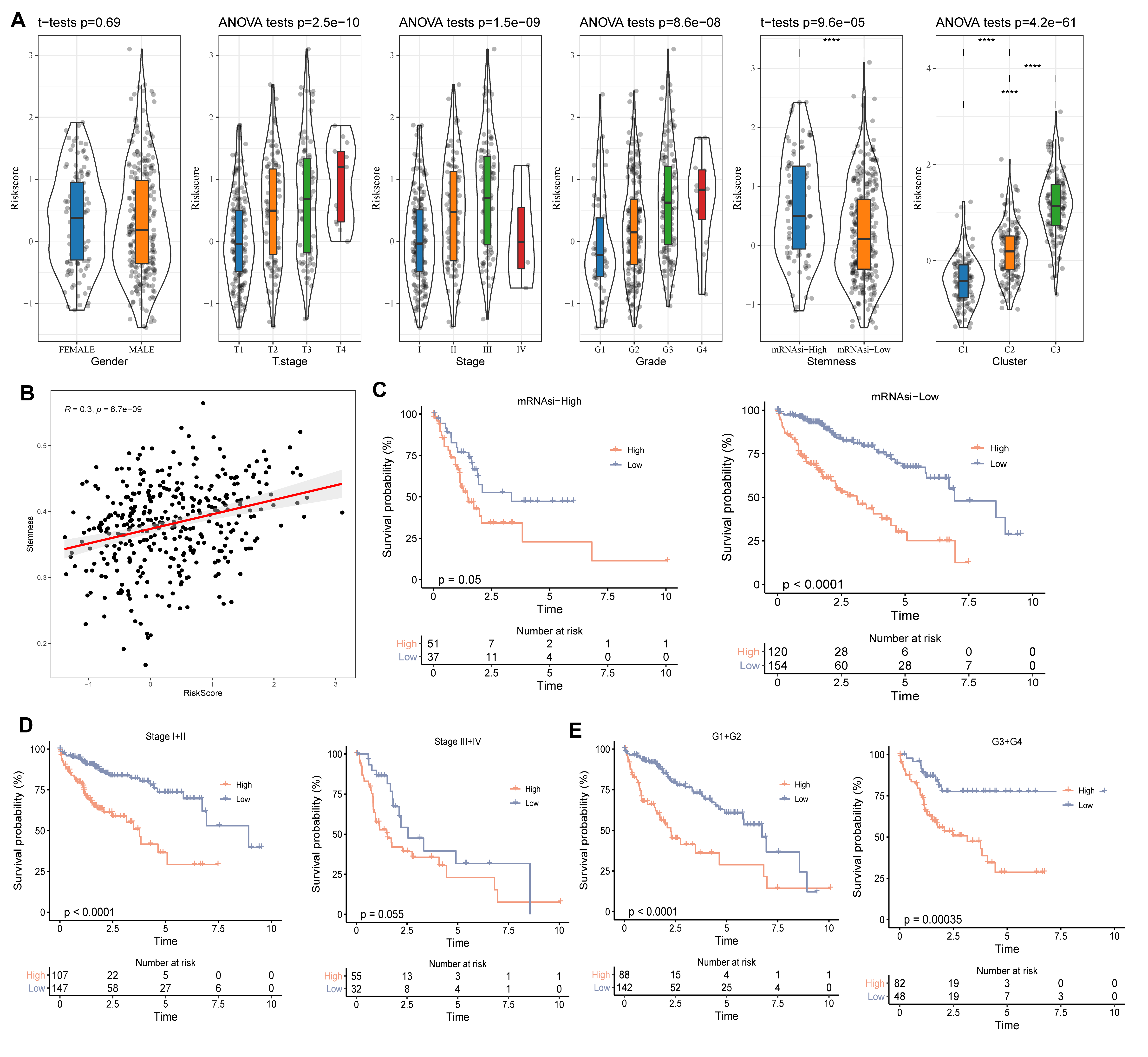

Supplement: Supplementary Figure 5 — Correlation between clinicopathological features and mRNAsi-related model. (A) An overview of the relationship between clinicalpathological features of HCC and riskscore. (B) In the TCGA-LIHC cohort, the relationship between riskscore and mRNAsi was analyzed. (C-E) The high-risk group in the TCGA-LIHC cohort had a poor prognosis among different stratified subgroups. ****P<0.0001. [file Image_5.tif]

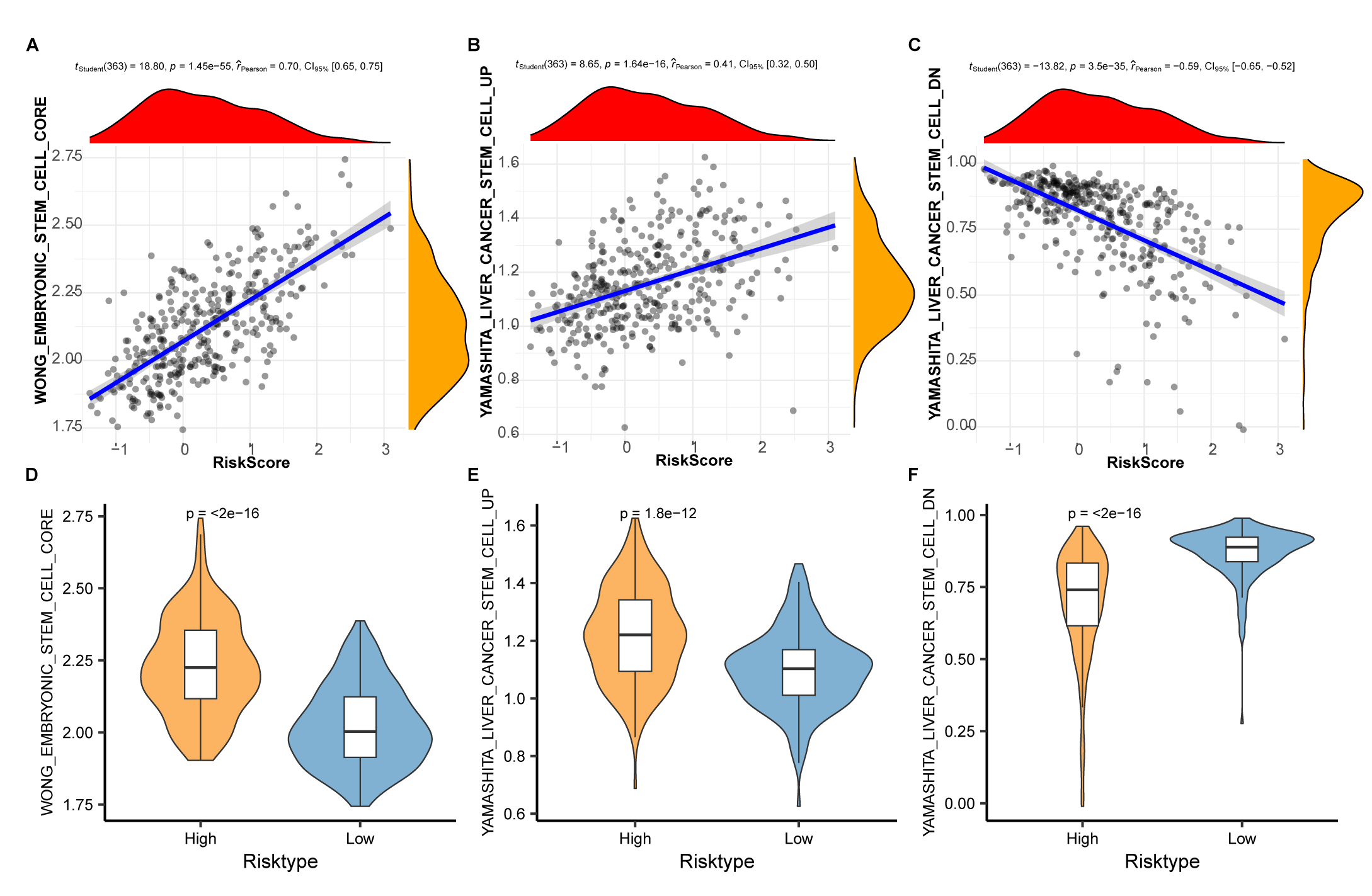

Supplement: Supplementary Figure 6 — The correlation between stemness-related gene sets and mRNAsi model. (A-C) Correlation analysis between the riskscore and wone embryonic stem cells core score and yamashita liver cancer stem cell up score, respectively. (D-F) The differences between high- and low- group in terms of wone embryonic stem cells core score, yamashita liver cancer stem cell up score, and yamashita liver cancer stem cell up score. [file Image_6.tif]
